# A visual approach to the economic evaluation of vaccines: opening the health economic black box

**DOI:** 10.1080/14737167.2021.1894931

**Published:** 2021-03-18

**Authors:** Enoch Kung, Maria Vittoria Bufali, Alec Morton

**Affiliations:** a Clinical Operational Research Unit, Department of Mathematics, University College London, London, UK; b Department of Management Science, University of Strathclyde, Glasgow, UK

**Keywords:** Economic evaluation, cost-effectiveness, meningitis, rotavirus, vaccination, vaccine, visualization

## Abstract

**Objectives:**

The economic evaluation of vaccines has attracted a great deal of controversy. In the academic literature, several vaccination advocates argue that the evaluation frame for vaccines should be expanded to give a more complete picture of their benefits. We seek to contribute to the debate and facilitate informed dialogue about vaccine assessment using visualization, as able to support both deliberation by technical committees about the substance of evaluation and communication of the underlying rationale to non-experts.

**Methods:**

We present two visualizations, an Individual Risk Plot (IRP), and a Population Impact Plot (PIP), both showing the beneficiary population on one axis and the degree of individual benefit and cost of an individual dose on the second axis. We sketch out such graphs for 10 vaccines belonging to the UK routine childhood immunization schedule and present our own analysis for the rotavirus and meningitis B vaccines.

**Results:**

While the IRPs help classify diseases by morbidity and mortality, the PIPs display the health and economic loss averted after introducing a vaccine, allowing further comparisons.

**Conclusion:**

The visualizations presented, albeit open to provide an increasingly complete accounting of the value of vaccination, ensure consistency of approach where comparative judgments are most needed.

## 1. Introduction

Vaccination has an enormously successful track record in public health. Diseases which were previously a scourge in even the most developed countries are now a distant memory. Vaccines can take credit for the elimination of smallpox and the near elimination of polio, two diseases which blighted the lives of millions and killed millions more throughout human history. Most commentators see the development of a vaccine as humanity’s best hope of permanently defeating Covid-19. More than any other technology, vaccines truly deserve to be designated as a miracle of modern medicine.

Nevertheless, not all vaccines merit deployment everywhere. This may be because of the local disease burden: there is no need for universal dengue vaccination in the west of Scotland, for example, as the climate is inhospitable to *Aedes aegypti* and *Aedes albopictus* which are the principal vector for this disease. However, it may also be because of constraints imposed by the local financing situation: lack of funding is a critical reason for the poor utilization of influenza vaccines in low- and middle-income countries. Thus, as with other technologies, vaccines are and should be subject to some form of economic evaluation, to determine whether they represent value for money for the funder.

The economic evaluation of vaccines, however, has also attracted controversy in both the scholarly and lay circles. There exists disagreement to what extent the social benefits of a proposed immunization should be factored into the decision-making. For example, a new immunization program benefits not only the recipients but to their families and carers, so there is an argument for broadening the scope of the analysis to cover these benefits [1]. Public perceptions of the benefits of vaccines are complex and labile, as highlighted by the phenomenon of widespread vaccine hesitancy but also by (at least in the UK) deep public concern about the lack of access to meningitis B immunization [2,3].

To contribute to the debate on the evaluation of vaccines, we propose a visualization that may facilitate informed dialogue among the various parties. Data visualization is a useful tool in healthcare decision-making by effectively distilling down data so that it can be digested both experts and the patients. Our visualization design carries sufficient data that can support both the deliberation by technical committees about the substance of the economic evaluation yet is simple enough for communication to non-expert stakeholders.

In this paper, we describe the design of the visualization and the data encoded in it. The core idea of our graph is to show the beneficiary population on one axis and the degree of individual benefit and cost of an individual dose, measured as QALY (Quality-Adjusted life years) loss on the second axis. We use the work by Panovska-Griffiths et al. [4] to generate the graphs for 10 vaccine-preventable diseases currently in the UK routine childhood immunization schedule and present our own analysis of the rotavirus and meningitis B vaccines. Furthermore, the visualizations which we present in this paper could be developed in several ways to incorporate many of the factors which other authors have suggested are important to take into account.

### 1.1. Background

Lately, several commentators have highlighted the limitations of current evaluation frameworks in accounting for the range of benefits flowing from immunization programs. Previous valuations, it is argued, failed to reflect the full magnitude of the overall health, social and economic outcomes of vaccines, and focused heavily on a narrow subset of factors, that present fewer methodological challenges and for which there is greater data availability. These are:

- Health gains: the reductions in morbidity and mortality yielded.
- Healthcare cost savings: the direct (medical and non-medical) costs averted for care providers and private citizens, due to fewer episodes of illness.
- Productivity gains (‘care-related’): indirect costs associated with the productivity losses that would have been incurred due to absenteeism (working or schooling days lost) by those seeking and providing care.

**Figure 1.**
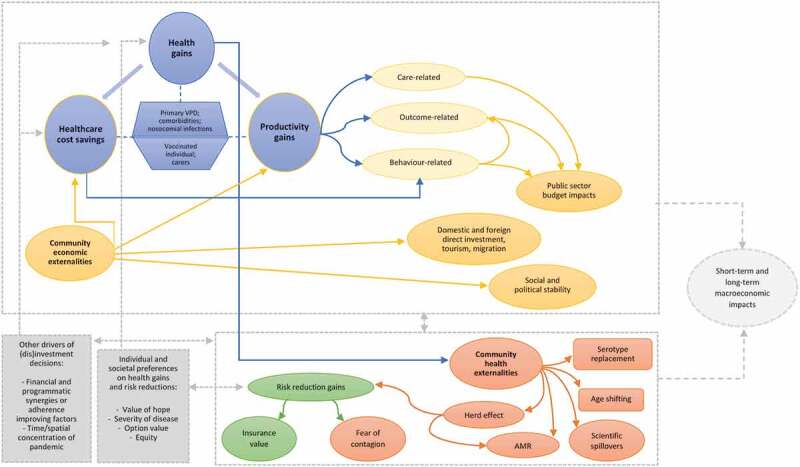
Flow chart of nonstandard factors in healthcare evaluation based on [5–9]

Drawing on several frameworks and lines of research [5–9], we provide a comprehensive taxonomy (Fig. 1) and description of these novel elements of value.

First, the scope of valuation efforts may be widened to include secondary health endpoints for vaccinated individuals, thus accounting for the reduced probability of developing comorbidities or nosocomial infections that may co-occur with the primary vaccine-preventable disease (VPD). So-called ‘health spill overs’, given that health-related consequences (e.g. physical or emotional strain, anxiety, grief) accompany productivity losses for those who take on caring responsibilities within the social network surrounding ill individuals, could also be considered.

Secondly, the evaluation frame could be extended to include ‘outcome-related’ and ‘behaviour-related’ productivity gains. The prevention, during childhood, of the long-term physical or neuro-cognitive impairments of numerous VPDs can enhance the wellbeing and human capital accumulation of an individual over the lifespan. Also, household resources may be devoted to meet the developmental needs of fewer children, who, being vaccinated, have greater survival chances. The resulting demographic dividend further may facilitate parental labor participation. Additionally, the consequent boost in economic growth and workforce productivity results in greater net transfers to public budgets. Finally, a country with higher vaccine coverage and reduced transmission rates of VPDs benefits from greater social and political stability, while better attracting flows of financial and human resources.

Third, compared to other health interventions, vaccination stands out for particularly striking health-related community externalities (or ‘ecological effects’). By limiting the occurrence and spread of VPDs, immunization schemes ensure: that unvaccinated individuals incur indirect protection, thanks to the health choices of those immunized (‘herd effects’); a reduction in the consumption of antibiotics and of the pace at which antimicrobial resistance (AMR) develops; and a reduction in risk and fear of being exposed to VPDs outbreaks. Individuals, whether immunized or not, may derive from vaccine protection greater peace of mind and gains in well-being due to reduced anxiety and worry. This factor can be particularly valuable to the society and partially operationalized in terms of ‘insurance value’ [9]. Furthermore, all the positive externalities yielded by advances in the field can be appropriated by those who have not directly contributed to them, such as future generations (‘scientific spill overs’). Conversely, there might be unintended negative externalities on population health too (shown in the square orange boxes), such as a raise in disease serotypes other than those covered by a vaccine and a shift in the age distribution of infection, to the expenses of age groups that might be more severely affected by infectious diseases.

Overall, the aforementioned factors jointly contribute to short- and long-term changes in macroeconomic conditions, by avoiding the shocks exerted by VPD outbreaks or shifting over time overall labor force participation and levels of productivity, health, well-being, and equity.

Finally, further complicating the broad picture, valuations and decision-making processes concerned with prioritization of immunization programs are required to account for how health preferences are informed by the value attached to definite health gains and risk reductions and to embed routine/crisis healthcare management considerations [8,9]. Empirical evaluations should embrace the possibility that the choices of individuals or entire communities are influenced by:

- Value of hope: individuals who value hope, as risk-takers, might prefer the intervention displaying higher uncertainty as regards the likelihood to gain an equal amount of health gains.
- Severity of disease: individuals might value differently equivalent gains in health if gained by preventing a single episode of highly severe illness, rather than multiple milder episodes.
- Option value: health gains being equal, individuals might prefer interventions that extend life, rather than improving its quality, to have greater chances to access more effective treatments and technologies yielded by possible future advances.
- Inequity aversion: according to the values endorsed at the societal level, funding decisions might embed the will to mitigate health, social and economic inequalities, to the benefit of age or socio-economic groups disproportionately burdened by VPDs.
- Catastrophe aversion: decision makers may have heightened concerns related to epidemic outbreaks that hit particularly harshly restricted geographical areas or escalate rapidly.

Having illustrated the components that populate this extended framework, we now describe the visualization approach developed. Remarks on how it can accommodate for a number of the nonstandard factors just outlined are drawn in the discussion.

## 2. Methods

This section describes the visualization. Developing from a basic model which captures individual impact of disease, we progressively add new pieces of information about the health impact, in terms of QALY loss, of the disease on the population as well as the economic impact of the immunization program. The sources of data which we use to generate the plots will also be described.

### 2.1. Visualization design

In this paper, we present two plots, an Individual Risk Plot (IRP) and a Population Impact Plot (PIP). The IRP communicates the QALY loss suffered by a non-fatal case of the disease in question along with the fatality rate of the disease. The summary plot consists of a horizontal red bar, with the height taking the number of QALY loss and a vertical blue bar, where the width represents the fatality rate, the x-axis being in the percentage of the population affected. The width of the red bar and the height of the blue bar will be used later to convey more information in the PIP. The plot illustrates the possible impact of the disease on the individual patient and also allows one to compare the health impact over different diseases by comparing the dimensions of the two bars.

The QALY losses for the diseases are calculated from global records of disability weights D [10,11] using the equation with a discount rate r of 3.5% [4,12–14] and t as the duration of the disease in question.

(1) $$QALYloss=\left(1-D\right)\frac{1-e^{-rt}}{r},$$

Beyond the individual health impact, it is important for the general public and decision makers to know the population health impact. To build the PIP, we change the x-axis to be the absolute number of patients annually, the width of the red bar to be the number of non-fatal cases, and the width of the blue bar to be the number of fatal cases. The Quality of Life lost will be expressed in days (QALD), instead of years (QALY) for reasons to be explained in a bit. The height of the blue bar is the QALD loss for a fatal case. The QALD loss must be calculated for the fatal cases using equation (1) where the duration is taken to be the difference between life expectancy and the estimated age of death from the disease.

Due to the difference in magnitude of how diseases affect the population, a log scale is used, as otherwise some bars would be too small to read.^1^ Another parameter that can be conveyed in this scale is the cumulative QALD loss for fatal and non-fatal cases suffered in the population. Prior to applying the log scale, this parameter is the area of the blue and red bar, respectively. The logarithm translates this parameter from an area to a length, by which we may compare the population QALD loss. This length is highlighted along the x-axis. The higher the cumulative QALD loss in the population, the longer this line would be. The Quality of Life is measured in days in order to generally ensure that we deal with positive values after taking logarithm. However, if one can be reasonably sure that QALY is greater than 1, then this conversion will be unnecessary, provided that any comparison between multiple diseases employs consistent units.

Information about costs of the immunization program is expressed in the lower right quadrant of the plot. We divide the costs of the vaccine into the product cost per one immunization course from the pharmaceutical company and the human costs of administering the disease. These are expressed by the height of a green and yellow bar, respectively. However, the existence of an immunization program also leads to a trade-off in the sense that the cost of the program can offset the future costs spent on the treatment of patients, including costs of GP visits, ambulances, hospitalizations, etc. One way to visualize the cost saved is to have a brown bar expressing the cost of treatment in height and number of cases averted in width.

In the results section, we generate IRPs with the basic design for a series of diseases based on data collected in [4]. We use the size of the red and blue bars to categorize a list of diseases according to their morbidity and mortality. The PIP is then generated for the diseases meningitis B and rotavirus.

### 2.2. Data sources

The data required in generating the plots are from a variety of government, academic, and business sources. For our initial plot design, we use the data listed in [4], the sources from which are listed in Table 1a. In our subsequent detailed visualization, we acquire the data on disease QALY loss, fatality rates, vaccine costs, the staff costs for administering vaccines, and the cost saved in the implementation of vaccines (see Table 1b). QALY loss for fatal cases are obtained by using equation (1), with the duration of diseases set as the difference between average life expectancy and estimated age of fatality. Priority of the sources is given to government records and then to reports and studies conducted in the UK. In absence of UK specific data, we refer to studies in high-income countries using the same vaccines as the UK. Assumptions made about QALY loss and healthcare costs are also listed (see Table 1c). Due to a lack of studies on QALY loss and associated parameters post-immunization for some diseases, some parameters will remain unchanged in our construction of the plots.

**Table 1a.** Sources of data used in basic plot for series of diseases in [3]

	Data	Country	Source	Reason of choosing
Diphtheria fatality rate	3.2%	St. Petersburg	[15]	Used in [3].
Diphtheria QALY loss	0.073	World	GBD [11]	
Mumps fatality rate	1.5%	Iran	[16]	
Mumps QALY loss	0.033	World	GBD [11]	
Tetanus fatality rate	11.5%	USA	[17]	
Tetanus QALY loss	0.0263	World	GBD [11]	
Rubella fatality rate	10%	USA	[18]	
Rubella QALY loss	0.0088	World	GBD [11]	
Polio fatality rate	10%	World	[19]	
Polio QALY loss	0.2022	World	GBD [11]	
Pneumococcal fatality rate	15%	UK	Vaccine [20]	
Pneumococcal QALY loss	0.0131	USA	CDC [21]	
Pertussis fatality rate	19.3%	USA	CDC [21]	
Pertussis QALY loss	0.0397	USA	GBD [11]	
Meningitis fatality rate	10.5%	USA	CDC [21]	
Meningitis QALY loss	0.0975	World	GBD [11]	
Measles fatality rate	0.355%	USA	CDC [21]	
Measles QALY loss	0.0195	World	GBD [11]	
Hib fatality rate	7.5%	USA	CDC [22]	
Hib QALY loss	0.0149	World	GBD [11]	
Rotavirus fatality rate	0.02%	UK	Department of Health [35]	Most suitable for study.
Rotavirus QALY loss	0.0027	Canada	[23,24]

**Table 1b.** Sources of data used in detailed plot on men B and rotavirus

	Country	Source	Reason of choosing
Men B Prevalence and Fatalities	UK	Public Health England [25]	Detailed record for number of cases and fatalities per year for the past decade including years before and after the introduction of the vaccine.
Men B QALY loss for non-fatal cases	Canada, Switzerland	H. Christensen et al. [26]	Men B QALY loss used in modeling impact of vaccine for UK using data from Canada and Switzerland, where the same vaccine is used.
Men B QALY loss for fatal cases	UK	Public Health England [3,25,27]	QALY loss is calculated by the formula in [3] with disability weight from [25] using the life expectancy recorded in [27]
Rotavirus Prevalence	UK	Public Health England [28]	Detailed record for number of cases per year for the past decade.
Rotavirus Fatalities pre vaccination	England and Wales	Department of Health [35] Jit et al. [29]	Jit et al. calculates the case-fatality ratio of rotavirus based on records in Hospital Episode Statistics due to difficulty in estimating rotavirus deaths. The government cites the study.
Rotavirus Fatalities post vaccination	UK	Public Health England [28]	PHE has access to data collected by the Health and Social Care Information Centre.
Rotavirus QALY loss for non-fatal cases	Canada	Jit et al. [30]	Rotavirus QALY loss used in modeling rotavirus vaccination.
Rotavirus QALY loss for fatal cases	UK	Public Health England [3,27,28]	QALY loss is calculated by the formula in [3] with disability weights in [35] using the life expectancy recorded in [24].
Men B cost of vaccine	UK	FiercePharma [33]	A report on the deal between Health Minister and pharmaceutical company GSK.
Rotavirus cost of vaccine	UK	GSK [34]	List price for vaccine per dose on pharmaceutical company GSK website.
Vaccine staff cost	UK	Mokiou [31]	The only study that focuses on vaccination administration costs.
Men B cost saved	UK	H. Christensen et al. [26]	The study lists the estimated public health response cost for a case of men B.
Rotavirus cost saved	UK	NIHR [32]	Study shows the reduction in number of GP, hospitals, and emergency departments and their corresponding savings.

**Table 1c.** Assumptions on QALY loss and vaccine costs

Assumptions
QALY losses are losses to people affected by the disease targeted by the vaccine
For a disease, the QALY loss of a single case, pre-vaccination and post-vaccination, remains the same, for both fatal and non-fatal cases.
The only costs relating to the vaccine and its implementation are healthcare costs.

## 3. Results

In this section, we give several examples of IRPs and PIPs. A series of IRPs is produced for a list of diseases, where the QALY losses and mortality rates can be found in Appendix B of [4]. These allow us to classify these diseases by morbidity and mortality. We then provide pre-vaccination and post-vaccination PIPs for the diseases rotavirus and meningitis B.

### 3.1. Plots

#### 3.1.1. Individual risk plots (IRPs)

**Figure 2.**
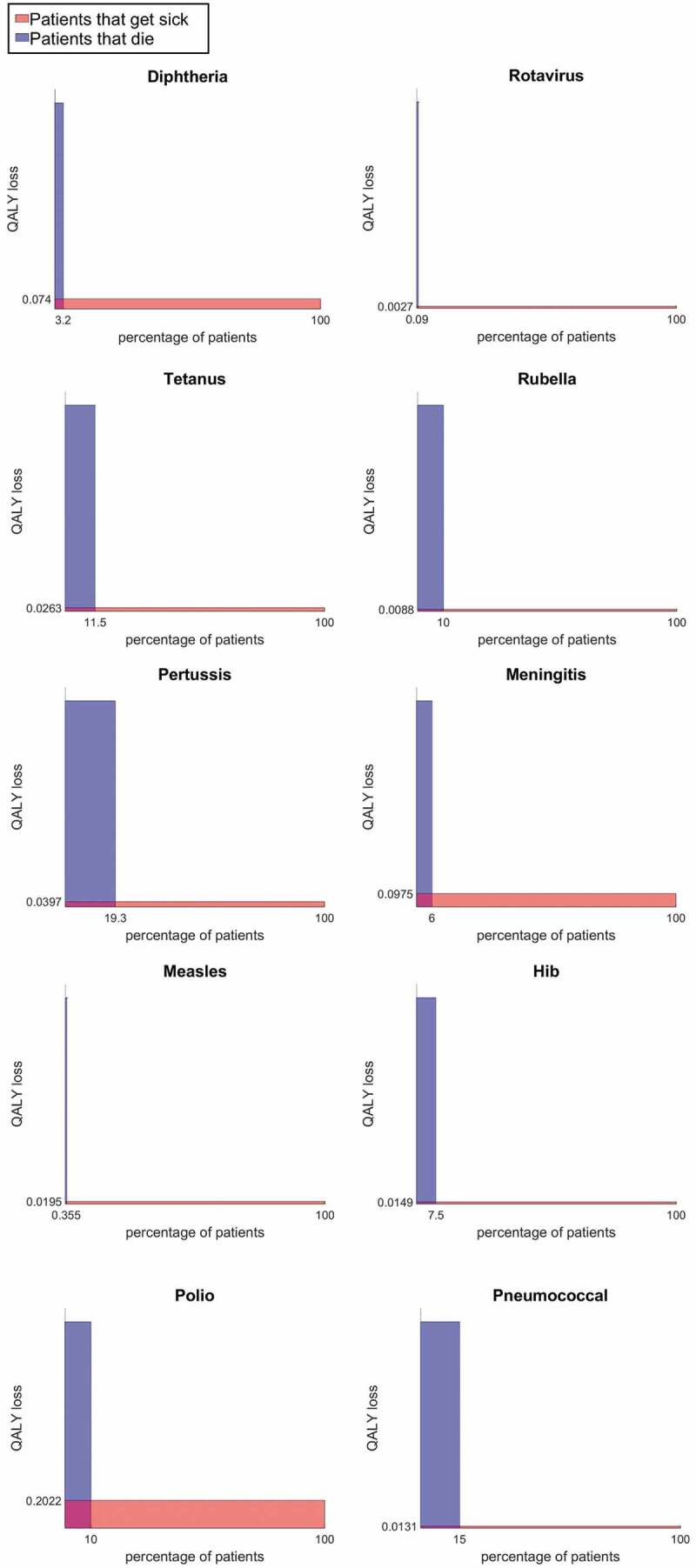
Individual Risk Plot (IRP) QALY loss for fatal and non-fatal cases for the following diseases: diphtheria, rotavirus, tetanus, rubella, pertussis, meningitis, measles, haemophilus influenza (hib), polio, pneumococcal

As presented in the IRPs (see Figure 2), we can easily see that how the profile varies for the different diseases. The red bar allows us to compare the damage done by the disease on surviving patients; polio and meningitis causes the most QALY loss for patients whereas rubella, hib and rotavirus affects patients the least. The height of the blue bar is roughly the same, indicating that the average age of death from the diseases is roughly the same; the one exception is pneumococcal disease for which death occurs with similar likelihood over different age groups compared to the others that mainly affect infants. The width of the blue bar compares the case-fatality rate of the disease, with tetanus and meningitis having the highest fatality.

##### 3.1.1.1. Morbidity and mortality

Using these plots, we can categorize the diseases according to morbidity and mortality, the former defined by the average QALY loss of surviving patients and the latter by the QALY loss of patients that die. Specifically, we can create four categories of high/low mortality against high/low morbidity as presented in the matrix below (Table 2).

**Table 2.** Categories of High/Low Morbidity and High/Low Mortality

		Morbidity	
		Low	High
Mortality	Low	Measles Rotavirus Haemophilus Influenza (Hib)	Diphtheria
	High	Polio Pneumococcal Tetanus Rubella Pertussis	Meningitis

Diseases with low mortality and morbidity are relatively unthreatening diseases from which most patients can easily recover from. High mortality but low morbidity can point to a disease that has effective treatment but access to treatment may be limited or time-sensitive. High morbidity, low mortality diseases are those that are not quite as deadly but take time to recover from or leaves the patient with lasting effects. The final category of high mortality, high morbidity are the most serious diseases.

#### 3.1.2. Rotavirus and Men B

**Figure 3.**
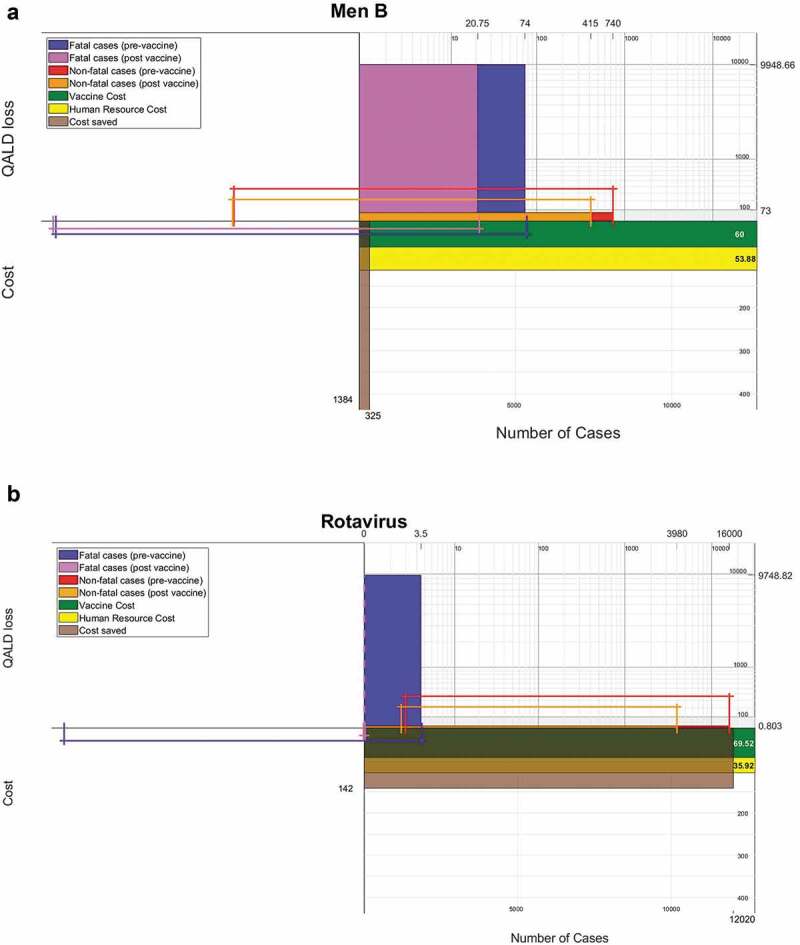
(A) Population Impact Plot (PIP) Health and cost impact of vaccination for meningitis B. (B) Population Impact Plot (PIP) Health and cost impact of vaccination for rotavirus

Our PIP displays (Figure 3) are demonstrated for patients suffering from rotavirus and those from meningitis B (henceforth, ‘men B’) before and after the introduction their respective vaccines. We focus on these two diseases because they occupy the two ends of the spectrum with respect to morbidity and mortality, as seen from the matrix.

To highlight the benefits of vaccination, we use a lighter purple and the orange to represent the post-vaccination QALD losses for fatal and non-fatal cases, respectively. These two bars are smaller compared to the pre-vaccination blue and red bars for both rotavirus and men B. In particular, for rotavirus, the fatality rate is zero, represented by the dotted purple line. Due to the lack of studies regarding QALD loss for both pre and post vaccination, the QALD losses are taken to be the same. Between the two diseases, men B has a higher fatality rate (wider blue and purple bars) and has a much greater impact in QALD loss for patients (taller red and orange bars), whereas rotavirus is more prevalent (longer red and orange bars).

With these added features, we can make several more comparisons. The highlighted intervals help us compare the population QALD loss for the two diseases, with a longer interval yielding a higher cumulative QALD loss. The intervals are calculated as logarithm of the area of the rectangle if the data were presented on a natural scale. The cumulative QALD loss obviously decreases (comparing intervals, purple and orange shorter than blue and red) after introducing the vaccine. Men B, due to a comparatively high fatality rate, inflicts a greater cumulative QALD loss than rotavirus for fatal cases (both purple and blue lines are longer for case of men B) and non-fatal cases (both orange and red lines are longer for case of men B) although men B is less prevalent. We remark here that when QALD loss does not change post-vaccination, which is true in our case, we can conclude that there is a greater proportional decrease in cumulative QALD in rotavirus due to a greater drop in cases. However, if QALD does change post-vaccination, we can compare the proportional decrease in cumulative QALD by comparing the length differences between intervals. For example, in non-fatal cases, the difference between the length of red and orange intervals is greater for rotavirus.

On the bottom right quadrant, we can compare the costs of vaccine production per course, which is three doses for men B at £20 per dose [33] and two for rotavirus at £34.76 per dose [34], and the administration cost. The similar size of the green bar means one immunization course costs roughly the same, while the human resource cost of administering the vaccine, due to the difference in doses, is more expensive for men B [35]. The human resource cost, however, is difficult to attribute to any single vaccine because multiple vaccines can be scheduled at one appointment. Men B also surpasses rotavirus in treatment cost saved per case averted, but the number of averted cases is lower; in total, the amount of treatment cost saved by the rotavirus vaccine is higher than that of men B. Due to the cost saved per case averted being much higher than that of rotavirus, we opted to not plot the entire bar in order to keep the green and yellow bars visible.

## 4. Discussion

Our approach shows many of the positive features highlighted by advocates of visualization tools [36]. The IRP and PIP designs allow users to make judgments on immunization programs as well as quick comparisons across diseases, without loss of the essential details such as the prevalence and costs. Hence, users can make informed decisions at the level of detail they choose to extract from them. Furthermore, the design is flexible in the way and amount of data presented. If comparing over multiple diseases, all comparisons are valid as long as the scales are kept consistent, which may be necessary to make the bars visible when dealing with diseases of low morbidity and/or mortality. Additionally, our designs are also customizable. In this respect, as long as reliable input data are available, our models easily lend themselves to the incorporation of additional data such as rates of GP visits, hospitalizations, or ER visits and their respective QALY losses, or saving items other than the direct healthcare costs averted, should such information be available. Also, provided that the budget holders’ remit is to allocate available resources to pursue socially desirable though not strictly health-related objectives, ‘care-related’ productivity gains accrued to immunized individuals and their potential carers could also be included. Similarly, in line with the goal of maximizing the entirety of population health, QALY losses potentially incurred by carers of infected individuals, if measured (e.g [5].), can be easily incorporated.

## 5. Expert opinion

We began the paper by noting that the evaluation framework for vaccines is disputed, and several commentators advocate for additional value dimensions to be included in the cost-effectiveness calculations. While it may be desirable to have a more complete accounting of the costs and benefits of vaccination, a major risk form this agenda is that the body of cost-effectiveness studies for vaccines become even less comparable than they already are. We believe that the most urgent challenge facing the research community, rather than trying to develop the most complete possible index of benefits for vaccine programs, is to develop a framework which surfaces the logic of the cost-effectiveness calculation and makes it possible to sense-check the assumptions and ensure broad consistency of approach where comparative judgments are required. Such a framework is what we have tried to develop in this paper.

True, we restrict in these visualizations our attention to the aggregate health burden imposed to the population due to mortality and morbidity. However, this information can serve as a building block for assessing the magnitude of many nonstandard impacts, where primary data is not available. For instance, the share of burden imposed on infants suggests what may be the potential long-term human capital loss or the health and productivity burden on their caregivers. Similarly, by knowing the distribution of health impacts on different population groups, concerns related to equity or severity of disease can be embedded in decision-making. Additionally, for quantifying the extra priority to be given to a certain vaccine under the effect of a spread of fear within a community, the models can support the elicitation of context-specific preferences, since a disease with a higher case fatality rate is presumably more fear-inducing than one where the health losses accrue over a large number of low-intensity episodes.

Current and forthcoming studies that seek to expand the scope of value assessments are surely deemed promising and worthwhile, as further insights into these novel elements of value can advance the understanding of the impacts of immunization programs and, ultimately, better inform their funding.

However, to date, accurate primary data for quantifying thoroughly certain factors are often sparse and unevenly available. Also, although fresh evaluation practices are being put forward (e.g [5].), they still remain narrowly applied, while asking for emergent methodological challenges to be tackled.

In light of the current limitations in data and methods, the pursuit of such broad-spectrum valuations risks not only to prove burdensome but also to prevent research from altering and impacting real-world policies as it could, since making it scarcely able to serve the primary function of allowing the prioritization of health spending decisions on the basis of comparisons. Provided that enlarging the array of impacts to factor into the decision-making would increase the complexity of modeling, we advocate that a promising area the scientific community should pay greater attention to is the development of models, as the ones here illustrated, that – albeit (or precisely because) essential – can guarantee that evidence-based immunization decision-making is extensively carried out, as well as widely and transparently communicated.

## 5. Conclusion

This paper demonstrates how a visual design can deliver the relevant information in a compact form, through two plots, IRP and PIP. While the design is open for future improvements, the approach shows promise in being an informative guide to both the public and to decision makers, both of whom are crucial for the future implementation and review of vaccination programs.

## Note

1. Along both the x and y-axes, any n is assigned to the length $log\left(1+n\right)$. The addition by 1 is so that zero is set at zero.

## Article highlights

- Economic evaluation of vaccines needs to encompass a more complete picture of the benefits of an immunization program
- Our visualization designs allow these benefits (social and economic) to be expressed and compared across different programs
- The visualizations are demonstrated on 10 vaccines, with emphasis on rotavirus and meningitis B
